# A Novel Muscarinic Antagonist R2HBJJ Inhibits Non-Small Cell Lung Cancer Cell Growth and Arrests the Cell Cycle in G0/G1

**DOI:** 10.1371/journal.pone.0053170

**Published:** 2012-12-28

**Authors:** Nan Hua, Xiaoli Wei, Xiaoyan Liu, Xiaoyun Ma, Xinhua He, Rengong Zhuo, Zhe Zhao, Liyun Wang, Haitao Yan, Bohua Zhong, Jianquan Zheng

**Affiliations:** Beijing Institute of Pharmacology and Toxicology, Beijing, China; Kyushu University, Japan

## Abstract

Lung cancers express the cholinergic autocrine loop, which facilitates the progression of cancer cells. The antagonists of mAChRs have been demonstrated to depress the growth of small cell lung cancers (SCLCs). In this study we intended to investigate the growth inhibitory effect of R2HBJJ, a novel muscarinic antagonist, on non-small cell lung cancer (NSCLC) cells and the possible mechanisms. The competitive binding assay revealed that R2HBJJ had a high affinity to M3 and M1 AChRs. R2HBJJ presented a strong anticholinergic activity on carbachol-induced contraction of guinea-pig trachea. R2HBJJ markedly suppressed the growth of NSCLC cells, such as H1299, H460 and H157. In H1299 cells, both R2HBJJ and its leading compound R2-PHC displayed significant anti-proliferative activity as M3 receptor antagonist darifenacin. Exogenous replenish of ACh could attenuate R2HBJJ-induced growth inhibition. Silencing M3 receptor or ChAT by specific-siRNAs resulted in a growth inhibition of 55.5% and 37.9% on H1299 cells 96 h post transfection, respectively. Further studies revealed that treatment with R2HBJJ arrested the cell cycle in G0/G1 by down-regulation of cyclin D1-CDK4/6-Rb. Therefore, the current study reveals that NSCLC cells express an autocrine and paracrine cholinergic system which stimulates the growth of NSCLC cells. R2HBJJ, as a novel mAChRs antagonist, can block the local cholinergic loop by antagonizing predominantly M3 receptors and inhibit NSCLC cell growth, which suggest that M3 receptor antagonist might be a potential chemotherapeutic regimen for NSCLC.

## Introduction

Lung cancer is the leading cause of cancer-related mortality worldwide and the number of cases and deaths related to lung cancer is on the rise in many parts of the world. Non-small cell lung carcinoma (NSCLC) accounts for nearly 80% of all cases of lung cancer. Despite aggressive efforts, treatments are unsatisfactory and survival rates remain dismal (<20%) [1]. Therefore, further understanding of the biology of NSCLC and development of novel therapeutic approaches for lung cancer treatment are needed.

Acetylcholine (ACh) is an important neurotransmitter in the central and peripheral nervous systems and plays key roles in learning, memory, autonomic control, and muscular contraction via activation of acetylcholine receptors (AChRs), including the muscarinic (mAChRs) and nicotinic receptors (nAChRs). Recently, it has been found that ACh is also widely synthesized by a variety of non-neuronal cell types, including airway epithelial cells [2], pulmonary pleura [3], small and large intestine, gall bladder, keratinocytes [4], glia [5], vascular endothelium [6] and most common cancer cells such as NSCLC, small cell lung carcinoma (SCLC), colon cancer, glial and ovarian carcinomas [7]. The widespread expression of non-neuronal acetylcholine is accompanied by the ubiquitous presence of choline acetyltransferase (ChAT), cholinesterase and receptors (nAChRs, mAChRs). Although the primary function elucidated incompletely, non-neuronal acetylcholine appears to be involved in the regulation of important cell functions, such as mitosis, trophic function, automaticity, locomotion, ciliary activity, cell-cell contact, cytoskeleton, as well as barrier and immune functions [7]–[9]. Therefore, the non-neuronal cholinergic system and acetylcholine, acting as a local autocrine and paracrine hormone, should be discriminated from the neuronal cholinergic system and neuronal acetylcholine.

Similarly, non-neuronal ACh stimulates cell growth through either muscarinic cholinergic or nicotinic cholinergic pathways. It has been reported that the growth of tumor cells was accelerated via activation of mAChRs in colon [10], lung [11]–[12], glial [13], and prostate [14]. In ovarian carcinomas, expression of mAChR correlates with a poor prognosis [15]. Song *et al* reported that interruption of autocrine muscarinic cholinergic signaling with M3 receptor antagonist 1,1-dimethyl-4-diphenylacetoxypiperidinium iodide (4-DAMP) or darifenacin has potential to inhibit SCLC cell growth both *in vitro* and *in vivo* [16]. In addition, many investigators have shown that activation of nAChRs by nicotine or its carcinogen derivative 4-(methylnitrosamino)-1-(3-pyridyl)-1-butanone (NNK) stimulated tumor cells growth in breast [17], lung [18], pleural mesothelioma [19], colon [20], bladder [21] and cervical cancer [22]. These studies have shown that a7 is the main nAChR subunit that mediates the proliferative effects of nicotine in cancer cells.

**Figure 1:**
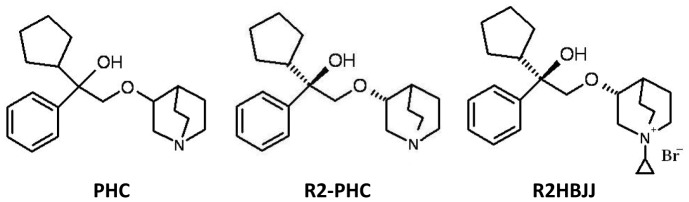
Structures of R2HBJJ and its parental compounds.

Penehyclidine hydrochloride (PHC) is an anti-cholinergic drug derived from hyoscyamine and developed independently by our institute. As its potential anticholinergic activity, it has been marketed for treatment of acute organophosphorus pesticides poisoning and chronic obstructive pulmonary disease (COPD) [23]–[24]. PHC is a racemic compound with two chiral centers, which compose four optical isomers (Fig. 1). Our previous work revealed that the isomer with R, R′-configuration (R2-PHC) displays the highest anticholinergic activity. To reduce the side effects by depressing access to the central nervous system, a new anticholinergic agent (R2HBJJ) is designed by introducing a quaternary ammonium salt group to R2-PHC molecule for treatment of peripheral diseases, such as lung cancers and COPD. In the present study, the selectivity of R2HBJJ for M1–M5 receptors is evaluated in detail and the antitumor activity of R2HBJJ on NSCLC cell lines *in vitro* and its potential mechanisms are explored. To our knowledge, this is the first study to investigate the effect of M3 antagonist on NSCLC cells.

## Results

### R2HBJJ displayed higher selective affinity to M_3_ and M_1_ receptor subtypes

**Figure 2:**
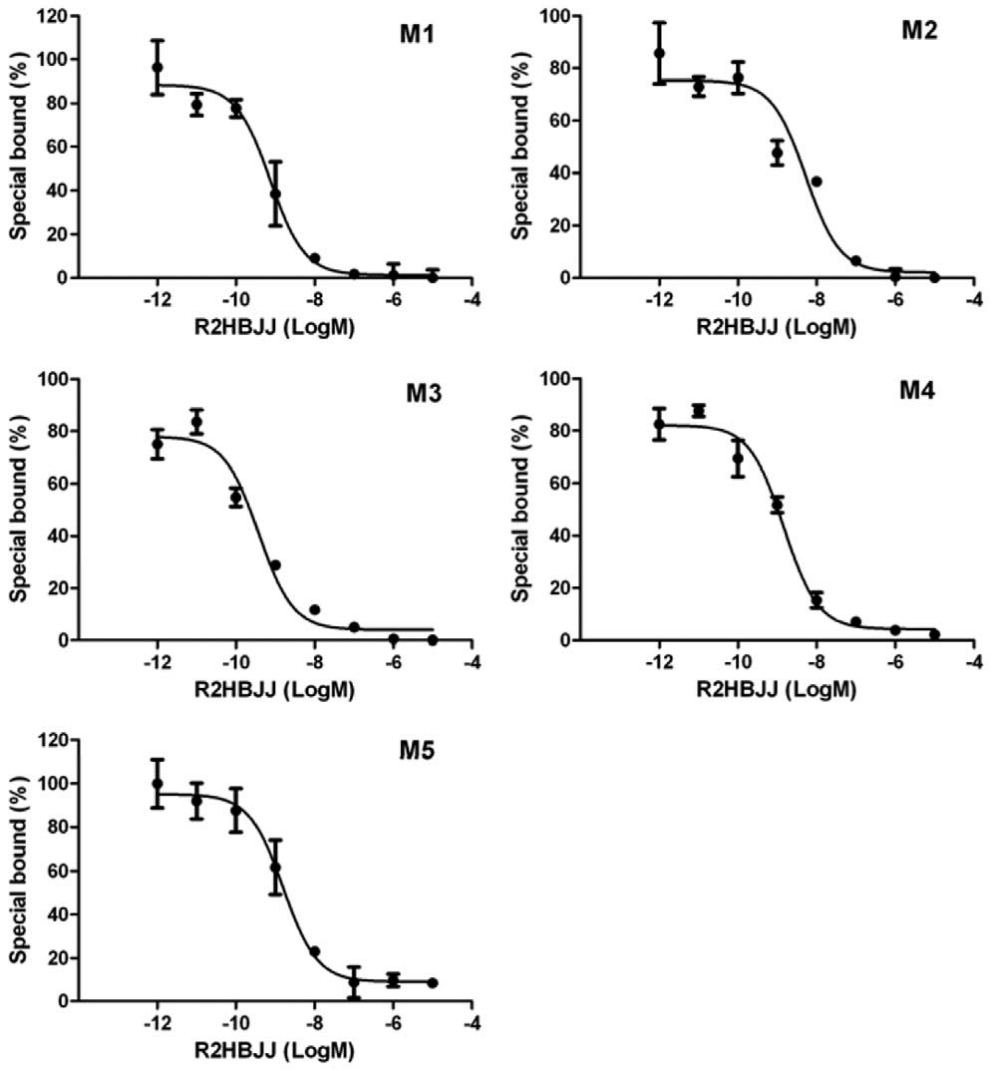
Competition of R2HBJJ with the binding of [^3^H] NMS to M1–M5 receptor subtypes. mAChRs proteins were incubated with [^3^H]-NMS at 37°C for 2 h in the absence and presence of increasing concentrations of R2HBJJ. Data represent the means ± SEM of three independent experiments performed in duplicate.

Saturation binding analysis yielded affinity binding constant (K_D_) values for [^3^H] NMS at human M1, M2, M3, M4 and M5 of 0.29±0.04, 0.81±0.17, 0.53±0.09, 0.19±0.03, and 0.48±0.13 nM, respectively. The relative selectivity for M1–M5 AChR subtypes of R2HBJJ was first determined at the level of receptor binding affinity in human mAChRs proteins (Fig. 2). The values of IC_50_ and K_i_ of R2HBJJ inhibiting [^3^H] NMS binding to M1–M5 receptor subtypes were summarized in Table 1. R2HBJJ exhibited a relative higher affinity to M3 and M1 receptor than M2 receptor. The rank of affinity of R2HBJJ for five different mAChRs was M3>M1>M4>M5>M2.

**Table 1:** Selective effects of R2HBJJ on muscarinic acetylcholine receptor subtypes (mol/L, mean ± SD, n = 3).

	M1	M2	M3	M4	M5
IC_50_	(7.39±1.45)×10^−10^	(5.33±2.89)×10^−9^	(3.80±2.63)×10^−10^	(1.47±1.89)×10^−9^	(1.61±2.37)×10^−9^
Ki	(7.86±3.39)×10^−11^	(1.33±0.52)×10^−9^	(6.79±3.71)×10^−11^	(1.06±0.12)×10^−10^	(2.64±0.98)×10^−10^

### R2HBJJ inhibited carbachol-induced tracheal contraction

**Figure 3:**
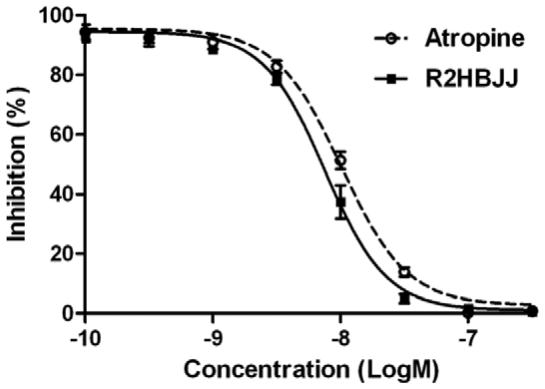
R2HBJJ inhibited the contractile response induced by carbachol in guinea-pig tracheal strip. Carbachol (1 µM) was used to induce contractile response. After that, the indicated concentrations of R2HBJJ or atropine were added cumulatively into the organ bath and the inhibitory concentration responses against carbachol-induced contraction were obtained. The values were expressed as percentages of the maximum contractile response in the absence of any antagonist. Each point represented the mean ± SD of five independent experiments.

To determine the blocking effect of R2HBJJ on mAChR, an ex vivo model of guinea-pig tracheal contraction was applied. The contraction of tracheal strip isolated from guinea-pig was induced by 1 µM of carbachol, a non-selective muscarinic agonist. And then, increasing concentrations of R2HBJJ (10^−10^–10^−6.5^ M) or atropine as a control antagonist, were added cumulatively into the organ bath. The inhibitory concentration response against carbachol-induced contraction was obtained. The results showed that R2HBJJ concentration-dependently inhibited the contractile responses of guinea-pig tracheae to the carbachol (Fig. 3). The 50% inhibition concentration (IC_50_) was 7.58±1.05 nM, which was statistical significantly less than that of atropine (10.21±1.04 nM, *P*<0.05). The data revealed that R2HBJJ exhibited a strong antagonistic activity on mAChRs expressed in guinea-pig trachea.

### NSCLC cell lines expressed cholinergic receptors and choline acetyltransferase

**Figure 4:**
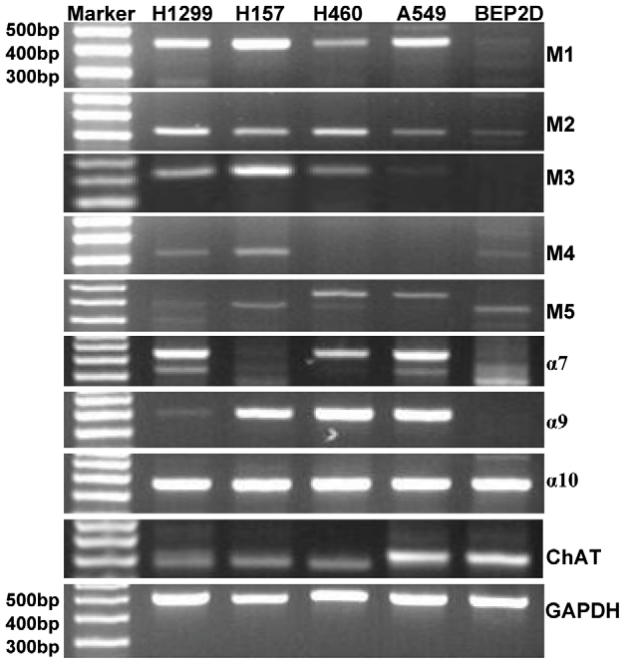
mRNA expressions of cholinergic receptors and choline acetyltransferase in NSCLC cell lines. RT-PCR was performed on total RNA prepared from the indicated cell lines. Primers were described in Table 3. GAPDH was used as loading control.

If a cholinergic autocrine loop is functional in NSCLC, then cells must express choline acetyltransferase (ChAT), mAChRs and/or nAChRs for ACh to provide autocrine cholinergic stimulation to NSCLC cell growth. To determine whether NSCLC cell lines express AChRs and ChAT, the mRNAs of five subtypes of mAChRs (M1–M5), three subtypes of nAChRs (α7, α9 and α10) and ChAT were examined by RT-PCR in human NSCLC cell lines H1299, H157, H460, A549 and human immortalized bronchi epithelial cell BEP2D. As shown in Fig. 4, the M1, M2, M5, α7, α9, α10 receptor subtypes and ChAT were almost expressed in all of the NSCLC cells tested. M3 receptor subtype was expressed in H1299, H157and H460, and was absent in A549. M4 receptor subtype was expressed in H1299 and H157, but not in H460 and A549. In contrast, human immortalized bronchi epithelial cell BEP2D expressed the mRNAs of different AChRs subtypes in lower levels, except for α10 subtype and ChAT. The results imply an acetylcholine autocrine loop existing in NSCLC cells.

### R2HBJJ inhibited NSCLC cell proliferation in vitro

**Figure 5:**
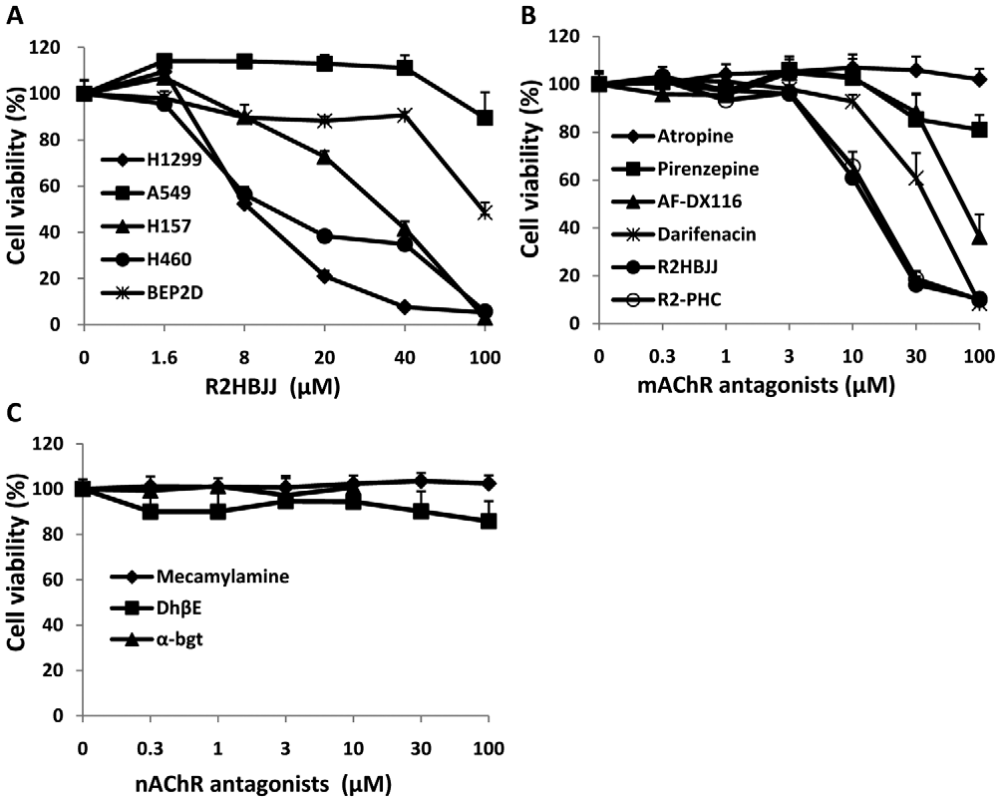
R2HBJJ and M3-mAChR antagonists inhibited NSCLC cell proliferation. Effects of R2HBJJ on human NSCLC cell lines and human immortalized bronchi epithelial cell BEP2D (A) and effects of mAChR and nAChR antagonists on H1299 cell lines (B and C, respectively). The cells were treated with the indicated concentrations of R2HBJJ or cholinergic receptor antagonists for 72 h. Cell viability was determined by SRB assay. Cells treated with solvent (DMSO) were used as a control, with viability set at 100%. Each data point represents the means ± SD of three independent experiments.

To evaluate the effect of R2HBJJ on cell growth of NSCLCs, the cells were treated with increasing concentrations of R2HBJJ (1.6–100 µM) for 72 h and cell viability was determined by using the sulforhodamine B (SRB) assay. The results showed that treatment with R2HBJJ markedly suppressed cell growth in a concentration-dependent manner in H1299, H460 and H157 cells, with a 50% growth-inhibitory concentration (GI_50_) of 8.5, 8.8 and 28.5 µM, respectively. In comparison, A549 and BEP2D cells were resistant to R2HBJJ, with a GI_50_ of more than 100 µM, the highest concentration tested (Fig. 5A). The data suggested that the anti-proliferative effect of R2HBJJ was dependent on the characteristics of cell lines and not the non-selective cytotoxicity.

Furthermore, the effects of other mAChRs (Fig. 5B) and nAChRs (Fig. 5C) antagonists on H1299 were observed. As shown in Fig. 5B and 5C, the known selective M3 mAChR antagonists darifenacin significantly inhibited H1299 cell proliferation in a concentration-dependent manner, whereas the non-selective mAChR antagonist atropine, non-selective nAChR antagonist mecamylamine, selective M1 mAChR antagonist pirenzepine, selective α4β2/α4β4 antagonist DhβE, and selective α7 antagonist α-bgt had no obvious effects on cell proliferation. The M2/M4-selective antagonist AF-DX116 also had inhibition on cell proliferation at the highest concentration tested. In comparison, R2HBJJ and its parental compound R2-PHC significantly inhibited H1299 cell proliferation in a concentration-dependent manner, which inhibitory potency was about 3.7-fold greater than that of darifenacin, the best one among the antagonists tested above. Because no exogenous AChR agonist was applied, the data revealed that endogenous ACh functioned as an autocrine growth factor signaling in human NSCLC cells, in part through activation of M3 receptor.

### Exogenous AChR agonist attenuated R2HBJJ-induced growth inhibition

To determine the correlation between cholinergic signal pathways and R2HBJJ-induced growth inhibition, exogenous AChR agonist ACh was used. The effects of ACh on the growth of H1299 and on R2HBJJ-induced growth inhibition were observed. It was found that exogenous ACh alone at 1, 10, and 100 µM for 72 h had no significant effects on H1299 cell growth (Table 2). R2HBJJ alone at 10 µM and 20 µM decreased the cell viability from 100±1.61% to 54.13±3.89% and 23.23±2.14% (n = 3), respectively. When ACh was administered prior to R2HBJJ treatment, the growth inhibition induced by R2HBJJ (both 10 and 20 µM) was atttenuated in the presence of exogenous ACh in a concentration dependent manner. Two-way analysis of variance (ANOVA) showed a siginificant effect of R2HBJJ treatment, ACh treatment, and R2HBJJ treatment×ACh treatment interaction (*P*<0.0001, respectively). Post-hoc tests confirmed that the following pretreatment concentrations of ACh, 10 µM and 100 µM, significantly attenuated the growth inhibition induced by 10 µM or 20 µM of R2HBJJ. When compared with R2HBJJ treatment alone at the level of 10 µM, 10 µM and 100 µM of ACh pretreatment increased the cell viability from 54.13±3.89% to 82.29±5.82% (*P*<0.01) and 91.1±3.65% (*P*<0.01), respectively. When compared with R2HBJJ treatment alone at the level of 20 µM, 10 µM and 100 µM of ACh pretreatment increased the cell viability from 23.23±2.14% to 35.57±4.48% (*P*<0.05) and 58.55±2.83% (*P*<0.01), respectively (Table 2).

**Table 2:** Exogenous replenish of ACh attenuated R2HBJJ-induced growth inhibition in H1299 cells (cell viability %, mean ± SD, n = 3).

R2HBJJ (µM)	ACh (µM)			
	0	1	10	100
0	100±1.61	101.71±1.80	104.74±2.74	99.05±2.72
10	54.13±3.89	55.04±6.79	82.29±5.82**	91.1±3.65**
20	23.23±2.14	24.1±1.94	35.57±4.48#	58.55±2.83##

Two-way ANOVA was used to compare the interaction of two treatment factors. Post-hoc analyses were performed with Bonferroni's tests.

** *P*<0.01, compared with the cells treated with 10 µM R2HBJJ alone.

# *P*<0.05,

## *P*<0.01, compared with the cells treated with 20 µM R2HBJJ alone.

### M3- and ChAT specific siRNAs decreased cancer cell proliferation

**Figure 6:**
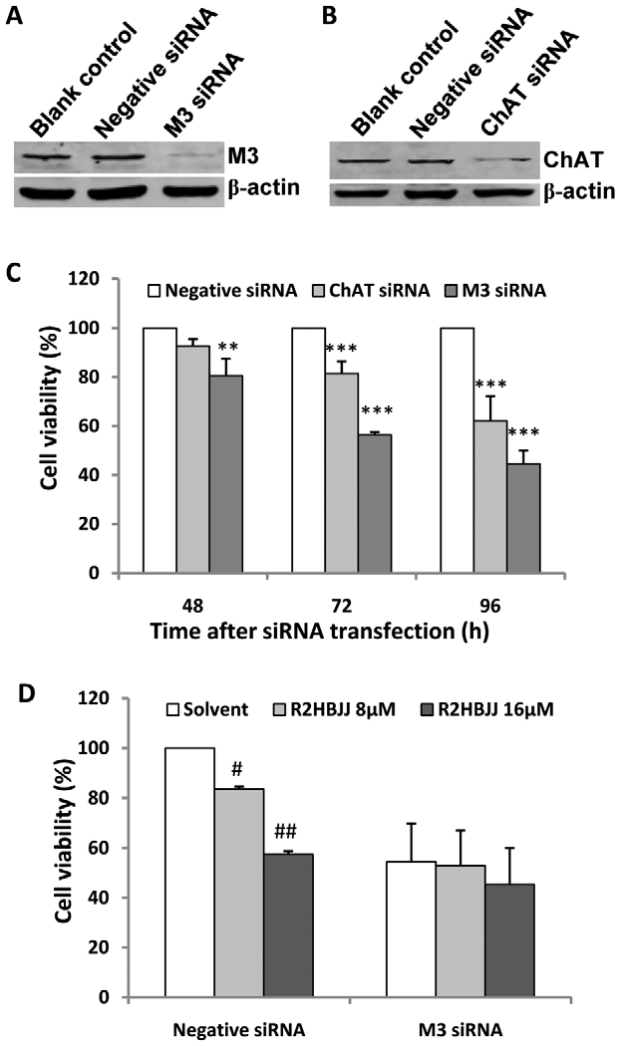
Effect of M3 or ChAT siRNA transfection on the growth of H1299 cells. Down-regulation of M3 and ChAT protein was detected by western blot 72 h post transfection of 200 nM siRNAs (A and B). Cells were incubated in the presence of 200 nM siRNA for 48, 72 and 96 h, after that, cell growth viability was determined using the SRB assay and compared with the mock control. ^**^ *P*<0.01, ^***^ *P*<0.001, compared with the negative control siRNA transfected cells at the same time point (C). Following M3 or Negative siRNA transfection, cells were treated with or without R2HBJJ (8 µM and 16 µM) for 72 h and the combination effect of M3 siRNA knockdown and R2HBJJ treatment on cell growth was investigated using the SRB assay. ^#^ *P*<0.05, ^##^ *P*<0.01, compared with the negative siRNA transfected cells treated with solvent. (D). Each data point represents the means ± SD of three independent experiments.

To further demonstrate that endogenous ACh and M3 receptor signal were involved in cell proliferation of human NSCLC cells, H1299 cells were transfected with M3- or ChAT-specific siRNAs and their proliferation was observed over time. Firstly, western blot analysis was used to identify the levels of these genes. As shown in Fig. 6A and 6B, M3 and ChAT protein levels were strongly decreased 72 h after transfection with targeting siRNAs compared to the negative control siRNA. By using SRB assay at 96-well plate, the potency of growth inhibition caused by silencing M3 or ChAT was tested at 48, 72 and 96 h post transfection. The result revealed that there was a time-dependent reduction of cell growth viability by M3- or ChAT-siRNA in H1299 cells compared to the negative control siRNA (Fig. 6C). The anti-proliferative effect of silencing M3 was visualized evidently early at 48 h post transfection (*P*<0.01) and the inhibition of 55.5% was achieved within a 96 h time frame (*P*<0.001). For ChAT-siRNA, its anti-proliferative effect was presented at 72 h post transfection (*P*<0.001), which was slower than M3 siRNA; and the inhibition of 37.9% was achieved 96 h post transfection compared to the negative control siRNA (Fig. 6C, *P*<0.001).

To further evaluate whether the anti-proliferative effect of R2HBJJ was M3 receptor specific, H1299 cells were treated with R2HBJJ after M3 siRNA transfection. The result showed that in negative siRNA treated cells, R2HBJJ exhibited significant cell growth inhibition, the cell viability were 83.5% (*P*<0.05) and 57.4% (*P*<0.01) after R2HBJJ 8 µM and 16 µM treatment. However, after the cells were treated with M3 siRNA, the cell viability was inhibited to 54.4%; R2HBJJ (8 µM and 16 µM) didn't further influence the cell growth, which suggested that silencing M3 receptor deprived of cellular sensitivity to R2HBJJ and accentuated the role of M3 in the effect of R2HBJJ (Fig. 6D).

### R2HBJJ arrested the cell cycle in G0/G1 phase

**Figure 7:**
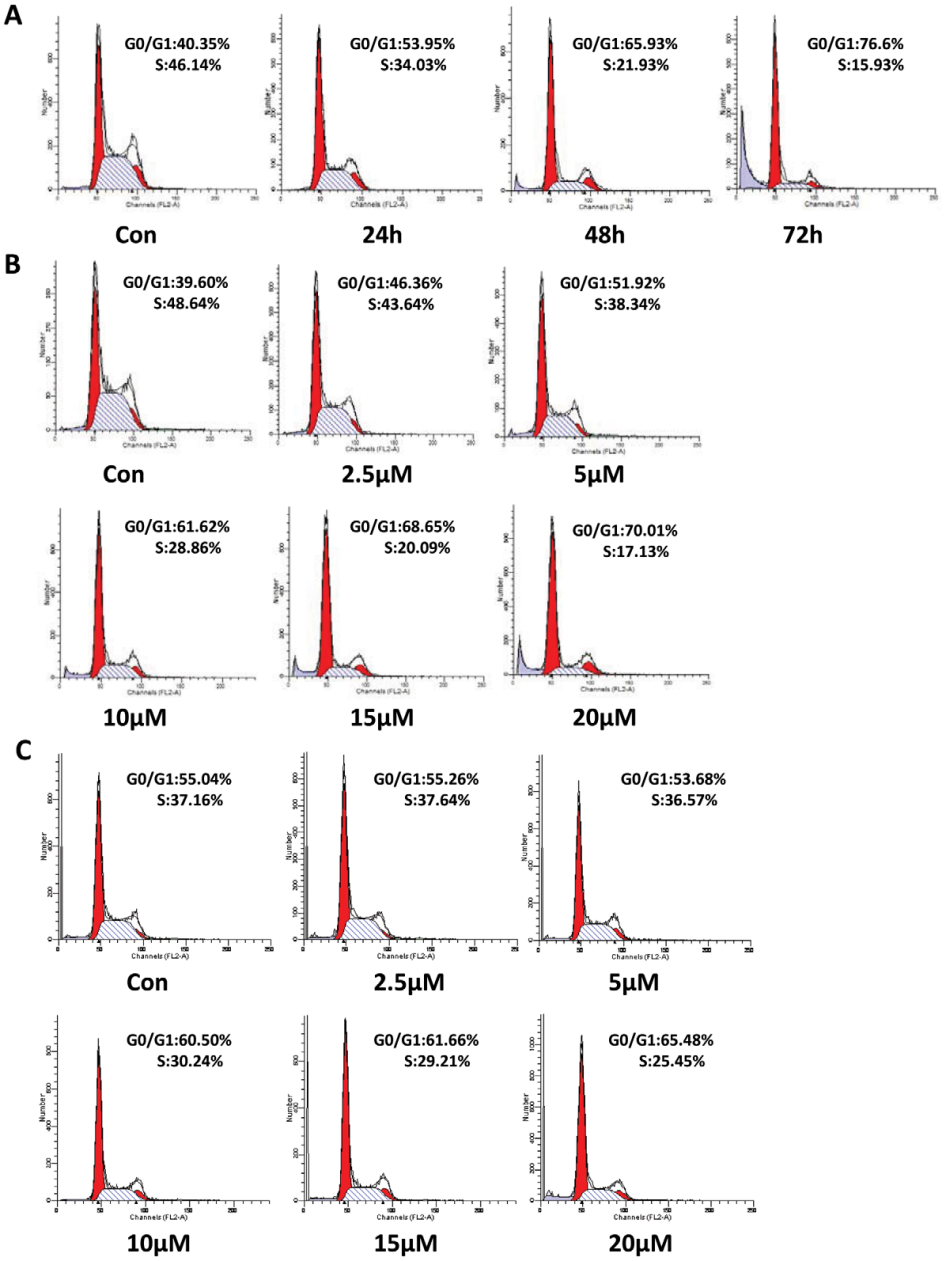
R2HBJJ induced G0/G1 Phase arrest on NSCLC cells. The cells were synchronized with 1% FBS for 24 h. After that, the cells were released into complete medium (10% FBS) containing 20 µM R2HBJJ for 24, 48, 72 h (A, H1299 cells) or various concentrations of R2HBJJ (2.5–20 µM) for 48 h (B, H1299 cells; C, H157 cells). Cells treated with solvent (DMSO) were used as a control. Cell cycle distribution was determined by using flow cytometric assay. The histograms displayed here were representative of three experiments.

To explore the potential mechanisms of R2HBJJ-induce cell death in NSCLC cells, the effects of R2HBJJ on cell cycle progression were observed by using the flow cytometric analysis. The results from H1299 cells showed that the percentage of G0/G1 phase increased from 40.35% to 76.60%, and that of S phase decreased from 46.14% to 15.93%, after treatment with 20 µM R2HBJJ for 72 h (Fig. 7A). Moreover, the H1299 cells treated with a range of R2HBJJ (2.5–20 µM) for 48 h also exhibited significant arrest in G0/G1 phase, with a percentage increase from 39.60% to 70.01% (Fig. 7B). In addition, similar result was obtained in H157 cell line, with a percentage increase in G0/G1 phase from 55.04% to 65.46% over concentration (Fig. 7C). The results indicated that R2HBJJ markedly suppressed the cell mitotic progression in time-dependent and concentration-dependent manners through arresting cells in G0/G1 phase and decreasing DNA synthesis.

### R2HBJJ down-regulated the levels of cell cycle regulatory proteins and Rb phosphorylation

**Figure 8:**
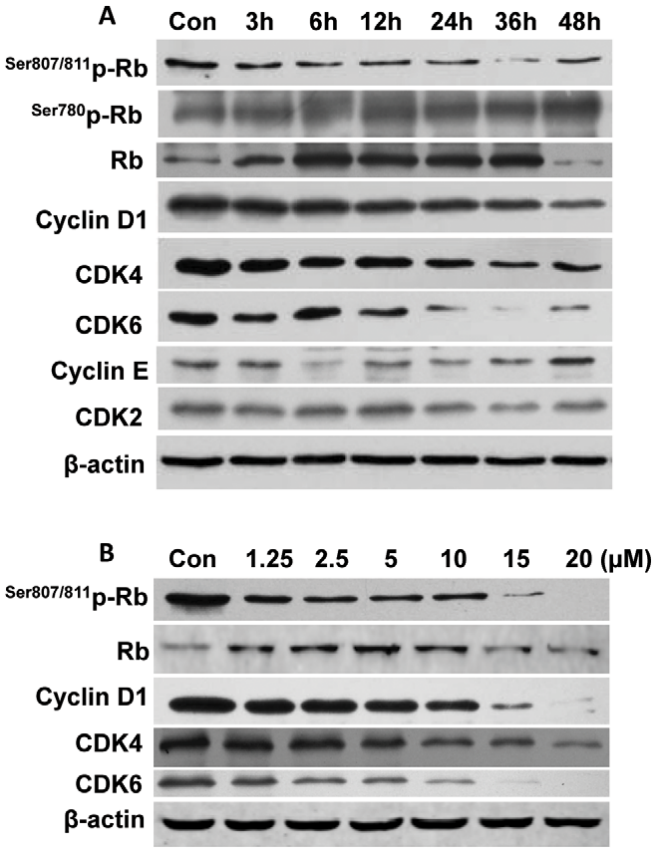
R2HBJJ down-regulated the levels of cell cycle regulatory proteins and Rb phosphorylation. H1299 cells were treated with 20 µM R2HBJJ for the indicated times (3–60 h, A) or with various concentrations of R2HBJJ (1.25–20 µM) for 48 h (B). Cell lysates were then subjected to western blot analysis. β-actin was used as the loading control.

Cell cycle transition from G1 to S is mainly activated by two different kinase composites: cyclin D1/CDK4, 6, and cyclin E, A/CDK2. Rb is the primary substrate of cyclin D-dependent kinase and plays a key role in the network of cell cycle regulation. To investigate the underlying molecular mechanisms of G0/G1 arrest induced by R2HBJJ, the time-dependent changes of several related cell cycle regulatory molecules were evaluated after treatment with 20 µM R2HBJJ in H1299 cells by using western blot analysis. As shown in Fig. 8A, treatment with R2HBJJ induced dramatic down-regulation of cyclin D1, CDK4 and CDK6 proteins time-dependently, whereas the levels of cyclin E and CDK2 remained basically unaltered during the same period. Moreover, the phosphorylation of Rb (particularly at Ser^807/811^) was decreased time-dependently. The decrease of Rb phosphorylated at the position of Ser^807/811^ occurred as early as 3 h after treatment and before apparent cell viability change. In contrast, the expression of basal Rb showed a significant persistent increase and then rapidly declined to an undetectable level at 48 h after treatment when cell growth suppression has been induced (data not shown), which may suggest a potential cellular adaptation to the decrease of phosphorylated Rb after R2HBJJ treatment. Further we evaluated the concentration response of Rb phosphorylation reduced after treatment with R2HBJJ for 48 h (Fig. 8B). The result showed that the decrease of Rb phosphorylated at Ser^807/811^ by R2HBJJ was concentration-dependent and occurred at the lowest concentration tested (1.25 µM). Consistently, the down-regulation of Cyclin D1, CDK4 and CDK6 proteins was observed in the cells treated by increasing concentrations of R2HBJJ. Similarly, the expression of basal Rb showed a significant increase over concentration at first and then rapidly declined to basal level. Together, these results suggested that Rb protein and the kinase composite of cyclin D1/CDK4, 6 were predominantly involved in the cellular G0/G1 arrest induced by R2HBJJ.

## Discussion

Many studies have demonstrated that ACh and other components of cholinergic signaling, including ChAT, cholinesterase, M and N cholinergic receptors, are present in a variety of non-neuronal tissues, including most common cancer cells such as NSCLC, SCLC, colon, glial, breast and ovarian carcinomas. In the local environment of tumors, concentrations of ACh at surface receptors may be even higher due to high cell densities in solid masses, increased secretion of ACh proximity to receptor location and decreased levels of cholinesterase in NSCLC [26], which implicates that cholinergic signaling may be further increased. The non-neuronal cholinergic system and ACh, as a local cellular growth signaling, plays a role in the development and progression of cancer and represents a potential new pathway to target tumor growth. Therefore, cholinergic antagonists interrupt the up-regulated autocrine signaling may provide a directed upstream approach to blocking proliferative pathway.

R2HBJJ, as a derivative of anticholinergic agent R2-PHC, was synthesized by our institute. In the present study, we evaluated the selectivity of R2HBJJ for M1–M5 muscarinic receptors and its anticholinergic activity. We also determined if interruption of endogenous cholinergic signaling with R2HBJJ has potential to inhibit NSCLC growth *in vitro*.

Our results demonstrated that R2HBJJ had significantly higher affinity for M3 and M1 receptor subtypes than M2 subtype, which suggested fewer M2 receptor-associated cardiovascular side effects. The rank of affinity of R2HBJJ for five different mAChRs was M3>M1>M4>M5>M2. The result is consistent with that obtained in its parental compound PHC before [27]. In the functional study, R2HBJJ inhibited the contractile responses of guinea-pig tracheae to the carbachol in a concentration-dependent manner. The inhibitory activity was stronger than that of mAChR antagonist, atropine.

Song *et al* reported that SCLC synthesize and secrete acetylcholine, which acts as an autocrine growth factor through both nicotinic and muscarinic cholinergic mechanisms [16]. Our study elucidated human NSCLC cell lines, like SCLC, express both M and N AChRs, as well as ChAT, implying that a functional foundation of cholinergic autocrine loop is present in NSCLC. Therefore, the growth inhibitory activity of R2HBJJ on several NSCLC cell lines, including H1299, H460 and H157 cells, may be mediated by both M and N cholinergic mechanisms. But when H1299 cells were treated with known selective or non-selective antagonists on M- and N- receptors *in vitro*, we found that only M3 receptor antagonist darifenacin presented a significant inhibition on cell growth. Meantime, our compounds R2HBJJ and R2-PHC also markedly inhibited the cell viability of H1299 cells, with a preferable effect than darifenacin. In the study of Song *et al*, treatment of SCLC cells with M3 receptor antagonists of 4-DAMP or darifenacin inhibited cell growth both *in vitro* and *in vivo*. Our results suggest that M3 receptor antagonists have inhibitory activity on NSCLC as well. The anti-proliferative effect of R2HBJJ on NSCLC may be related to its selective M3 receptor antagonistic activity. Russo P *et al* reported that inhibition of α7-nAChR with a powerful high affinity antagonist α-CbT induced antitumor activity in NSCLC and pleural mesothelioma by triggering apoptosis [18]–[19]. However, in our investigation, another α7-nAChR antagonist α-bgt was used to treat H1299 cell and the effect on cell viability was not observed, which suggested that α7-nAChR signaling may be not the primary mediator in H1299 cell growth.

In the present study, we failed to observe the proliferative effect of exogenous ACh on H1299 cells, which may imply that endogenous ACh has provided enough stimulant effect on cell proliferation. However, exogenous replenish of ACh is needed to antagonize competitively the inhibition of R2HBJJ on H1299 cells. The result stressed that the effect of R2HBJJ on H1299 cell growth was achieved via impacting on endogenous ACh signaling. In the present study, ChAT siRNA was proved to inhibit cell growth, which further supported this hypothesis. Moreover, it is noteworthy that treatment with M3 receptor siRNA resulted in a significant growth inhibition on H1299 cells when compared with negative control siRNA treated cells. The potency of inhibition achieved 50% or more within a 96 h time frame. When M3 receptor was effectively knocked down in H1299 cells, the cells lossed their sensitivity to R2HBJJ. The results further accentuated the role of M3 in the anti-proliferative effect of R2HBJJ.

Cell cycle arrest is a major mechanism preventing tumor growth. Our results from flow cytometric analysis showed that R2HBJJ markedly suppressed the cell mitotic progression through arresting cells in G0/G1 phase. Cell cycle transition from G1 to S controls DNA synthesis and requires hyperphosphorylation of a tumor suppressor, Rb [28]. In the cycle process, catalytic activity of cyclin D1 associating with CDK4/6 is first manifested by mid-G1, increases to a maximum at the G1/S transition and contributes to G1 exit. Whereas cyclin E and cyclin A associating with CDK2 is activated at the G1/S transition or in the early S phase, respectively. Phosphorylation of Rb is initially triggered by active Cyclin D1-CDK4/6 complexes and later accelerated by Cyclin E-CDK2. This phosphorylation allows the dissociation of transcription factors, such as E2 promoter-binding factor (E2F) and the proto-oncogene c-Abl overexpressed or mutated in a number of malignant tumors, which can transactivate S-phase genes encoding for proteins that amplify the G1-to-S phase switch and are required for DNA replication [29]. Of the cell cycle proteins examined in the present study, the expressions of cyclin D1, CDK4 and CDK6 proteins were down-regulated time-dependently, whereas the levels of cyclin E and CDK2 were not affected significantly. Moreover, the phosphorylation of Rb at the position of Ser^807/811^ was inhibited by R2HBJJ time-dependently, which could abolish the interaction between c-Abl and Rb and retard the activation of c-Abl and the cell cycle progression [30]. Together, our data suggested that Rb protein and the kinase composite of cyclin D1/CDK4, 6 were predominantly involved in the cellular G0/G1 arrest induced by R2HBJJ. The results also suggested that R2HBJJ exerted its retarding effect at the early phase of G0/G1.

In fact, regulatory molecules governing early cell cycle progression such as cyclin D1-CDK4/6-Rb are the frequent targets of genetic alterations in an exceptionally high percentage of lung cancers [31]. Many studies have demonstrated that strategies to target proteins associated with early cell cycle progression and the G1 restriction point may be useful in anticancer therapy [32]–[33]. Cyclin D1 has also been shown to be a downstream target of several signal transduction pathways mediated by such oncogenes as Neu, Ras, and β-catenin. A recent study supports the promise of co-targeting Cyclin D1 as a predictive and personalizing approach for lung cancer prevention and therapy [34]. Furthermore, ACh acting through mAChR has been shown to lead to cell proliferation by activation of MAPK, ERK1/2 and regulation of cell cycle progression [11], [16]. Our unpublished data also showed that early apoptosis was involved in the anti-proliferative effect of R2HBJJ, but the underlying mechanisms remained to be further explored.

In conclusion, the current study reveals that NSCLC cells express an autocrine and paracrine cholinergic system which stimulates the growth of NSCLC cells. R2HBJJ, a novel muscarinic antagonist, can block the local cholinergic loop by antagonizing predominantly M3 receptors and inhibit NSCLC cell growth. The mechanism for R2HBJJ-induced cell cycle G0/G1 arrest involves down-regulation of cyclin D1-CDK4/6-Rb. The results suggest that M3 receptor antagonists possibly become a new potent chemotherapeutic regimen for NSCLC.

## Materials and Methods

### Materials

Radiolabeled compound [^3^H] N-methylscopolamine ([^3^H] NMS), human muscarinic receptor (M1, M2, M3, M4 and M5) proteins and BetaPlate Scint of membrane were obtained from Perkin Elmer Life and Analytical Sciences. R2-PHC and R2HBJJ (chemical structures shown in Fig. 1) were synthesized by our institute. The chemical structures were confirmed by nuclear magnetic resonance spectrum analysis. Atropine, pirenzepine, mecamylamine, α-bungarotoxin (α-bgt), carbachol, and ACh were purchased from Sigma. Dihydro-β-erythroidine hydrobromide (DhβE) and AF-DX116 were purchased from Tocris. Darifenacin was purchased from Beijing Huafeng United Technology. Antibodies used for western blotting were purchased from Cell Signaling for retinoblastoma tumor suppressor protein (Rb), phospho-Rb (Ser^780^ and Ser^807/811^), Cyclin D1, Cyclin E, CDK2, CDK4, CDK6. M3-AChR was obtained from Santa Cruz. ChAT, β-actin, goat anti-rabbit and goat anti-mouse immunoglobulin labeled with horseradish peroxidase were purchased from Beijing Zhongshan Jinqiao Biotechnology.

### Radioligand Binding Assay

Saturation binding was performed using a range of concentration of [^3^H] NMS (0.3–19.5 nM; 82 Ci/mmol) in the absence and presence of atropine (500 µM) to determine nonspecific binding. [^3^H] NMS, mAChRs proteins and R2HBJJ were individually diluted with the binding buffer, which consisted of 110 mM NaCl, 5.3 mM KCl, 1 mM MgCl_2_, 1.8 mM CaCl_2_, 25 mM glucose, 58 mM sucrose, 20 mM HEPES (pH 7.4). Binding assays were performed in a final volume of 0.5 ml containing 18 to 42 µg of receptor protein and were incubated for 2 h at 37°C. Competition binding assays were performed using 2.4 nM of [^3^H] NMS in the absence and presence of a range of antagonist concentrations (10^−6^ - 10^−13^ M). Bound radioligand was separated from free by rapid vacuum filtration through GF/C glass fiber filters (Whatman). The filters were rinsed three times with ice cold PBS. After being dried, the fibers were placed in scintillation vials with 0.5 ml of BetaPlate Scint. Radioactivity trapped on the filters was quantified by a BECKMAN Beta counter. The assay was repeated three times with duplicate samples.

### Guinea-pig tracheal contraction

Female guinea-pigs (350–450 g) were used in this study, which were purchased from Beijing Animal Center. Animal experiments were carried out in accordance with the NIH Guidelines for the Care and Use of Laboratory Animals (1996) and the institutional guidelines of Beijing Institute of Pharmacology and Toxicology. The experimental protocol was approved by the Animal Research Advisory Committee of Beijing Institute of Pharmacology and Toxicology. The experiment was performed as described previously [25]. The animals were killed by a blow to the head. The trachea was excised, cleaned of adhering fat and connective tissue, cut into 2–3 rings, each containing about two cartilages. Each ring was then opened by a longitudinal cut, forming a tracheal chain with the smooth muscle in the center and the cartilaginous portion on the edges. The segments were mounted in an organ bath containing 20 ml Krebs-Henseleit (KH) solution of the following composition (mM): NaCl 118.0, KCl 4.7, CaCl_2_ 2.52, MgSO_4_ 1.2, NaHCO_3_ 25.0, KH_2_PO_4_ 1.2, and glucose 11.0. The tissue bath solution was maintained at 37°C and gassed with 95% O_2_ plus 5% CO_2_. Tracheal preparations were equilibrated in the medium for 60 min with four changes of KH solution and maintained under an optimal tension of 1.5 g before specific experiment protocols were initiated. Segments were first pre-contracted with 1 µM carbachol for 10 min till reaching stable plateaus, then washed completely until the segments reached basal tension and maintained in KH solution for another 60 min with four changes. Thereafter, segments were incubated with 1 µM carbachol for 10 min again and the experiments were started with this preparation. R2HBJJ or atropine was then administered cumulatively to produce their concentration-response curves. Results are expressed as percentages of the maximal response for carbachol before the addition of R2HBJJ or atropine. Contractions were recorded isometrically via a force-displacement transducer (SQG-4, Chengdu Scientific Instrument, China) connected to a Four-channel Oscillograph (Yimei Biotechnology, Nanjing, China).

### Cell Lines and Cell Culture

Human NSCLC cells H1299, H157, H460 and A549 were commercially obtained from the Cell Resource Center, IBMS, CAMS/PUMC (Beijing, China). The human papillomavirus (HPV18) immortalized bronchi epithelial cell BEP2D was developed by the research group of Dr. Curtis C. Harris (Division of Cancer Etiology, National Cancer Institute, NIH) [35]. Under the generous permission of Dr. Harris, we received the cell line as a gift from Dr. Tom K. Hei (Center for Radiological Research, Columbia University) [36]. H1299, H157 and H460 were routinely cultured in RPMI-1640 medium (Sigma) supplemented with 10% fetal bovine serum (FBS). A549 was maintained in Dulbecco's modified Eagle's medium (Hyclone) supplemented with 10% FBS. BEP2D was maintained in serum-free LHC-8 medium (Invitrogen) supplemented with epidermal growth factor and other growth supplements as described previously [35]–[36]. Cells were cultured at 37°C in a humidified incubator containing 5% CO2.

### RT-PCR

Total RNA was extracted from cells using TRIzol reagent (Invitrogen). 1 µg aliquot of each RNA sample was reverse-transcribed to synthesize cDNA in a 20 µl reaction volume by using an AMV-RT kit (Promega). Incubate the reaction using Oligo (dT)_15_ Primer at 42°C for 60 min, 95°C for 5 min, 4°C for 5 min. PCR was carried out in 25 µl of a reaction mixture with the following conditions: 95°C for 5 min, 35 cycles at 94°C for 30 s, 55°C for 1 min, and 72°C for 1 min. The primers used for PCR were shown in Table 3. GAPDH was used as the loading control. The PCR products were resolved on a 1.5% agarose gel stained with ethidium bromide.

**Table 3:** Primers used for RT-PCR.

Gene	Primers	Length (bp)
M1	S: 5′-TGGCTGCCTTCTACCTCC-3′ A: 5′-CTCGATCACGCCCTTTCT-3′	429
M2	S: 5′-ACCATTATCGGGAACATC-3′ A: 5′-CATCATACCTGCCATTTT-3′	321
M3	S: 5′-ACCGTCACTCATTTCG-3′ A: 5′-GTTGTTCGTTTGGCTC-3′	449
M4	S: 5′-TATGAGACGGTGGAAATGGT-3′ A: 5′-GCAGAAGTAGCGGTCAAAGC-3′	321
M5	S: 5′-ATCATGCCCTGCCCCTTCC-3′ A: 5′-GTAGCTTGCTGTTCCCCTGCC-3′	391
α7	S: 5′-GCCGCAGGACGCTCTACTAT-3′ A: 5′-ACGGCGCTCATCTCCACACT-3′	442
α9	S: 5′-GTCCAGGGTCTTGTTTGT-3′ A: 5′-ATCCGCTCTTGCTATGAT-3′	403
α10	S: 5′-CTGTTCCGTGACCTCTTT-3′ A: 5′-GGAAGGCTGCTACATCCA-3′	388
ChAT	S: 5′-GGAGATGTTCTGCTGCTATG-3′ A: 5′-GGAGGTGAAACCTAGTGGCA-3′	280
GAPDH	S: 5′-AGGTCGGAGTCAACGGATTTG-3′ A: 5′-GTGATGGCATGGACTGTGGT-3′	532

### Cell Viability Assay

Cells were seeded at a density of 6.0×10^3^ cells/well in 96-well plates. After overnight incubation, the cells were treated with R2HBJJ (1.6–100 µM) or other antagonists (0.3–100 µM) for 72 h. The inhibitory effects of the antagonists on cell growth were determined using SRB assay, as described previously [37]. For the combination administration, the agonist was added 1 h before R2HBJJ. Each experiment was performed in six replicates and repeated for three times. Cells treated with solvent (DMSO) were used as a control, with viability set at 100%. The 50% growth-inhibitory concentration (GI_50_) was calculated with Hill equation after curve fitting by GraphPad Prism 5 software.

### Small interfering RNA transfection and assay

The M3 receptor and ChAT specific small interfering RNA (siRNA), the glyceraldehyde-3-phosphate dehydrogenase (GAPDH) positive control siRNA (used in preliminary experiments to optimize the siRNA transfection efficiency, data not shown), the negative control siRNA were designed and synthesized by Guangzhou Ruibo Biotechnology (Guangzhou, China). The target sequences for M3 receptor and ChAT are 5′-CCTGGTAATTGTGTCATTT-3′and 5′-GCAAGCCATTGGCAACAAA-3′, respectively. The negative control siRNA consists of a proprietary siRNA sequence not corresponding to any eukaryotic gene.

H1299 cells were seeded at 1×10^5^/well in six-well plates one day prior to transfection and 1.5 mL of the medium without antibiotics was added to each well. The siRNA were transfected at a concentration 200 nM each in serum-free Opti-MEM I medium with Lipofectamine™ 2000 reagent (Invitrogen) according to the manufacture's instruction. Following 6 h incubation, the transfection medium was replaced with RPMI-1640 containing 10% (v/v) FBS. 72 h after transfection, the cells were harvested for Western blotting analysis. For cell proliferation assay, H1299 cells were seeded at 3.5×10^3^/well in 96-well plates one day prior to transfection. 48 h, 72 h and 96 h after transfection, cell growth viability was respectively determined using SRB assay. An independent experiment was performed to observe the combination effect of M3 siRNA knockdown and R2HBJJ treatment. While the transfection medium was replaced with complete RPMI-1640 medium 6 h after transfection, cells were treated with or without R2HBJJ (8 µM and 16 µM) for 72 h. Each experiment was performed in four replicates and repeated for three times. Cell viability of negative control siRNA was set at 100%.

### Cell Cycle Analysis

Alterations in cell cycle were determined by using flow cytometric assay. Cells were seeded at a density of 1×10^5^ cells/well in six-well plates and synchronized by plating in RPMI-1640 containing 1% FBS for 24 h. For time-dependent response, cells were incubated in 2 ml fresh RPMI-1640 containing 10% FBS with 20 µM R2HBJJ for 24, 48, 72 h. For concentration-dependent response, cells were incubated for 48 h with R2HBJJ at final concentrations of 2.5, 5, 10, 15, 20 µM in 2 ml fresh RPMI-1640 containing 10% FBS. Fresh media added in the control well simultaneously. After treatment, the cells were harvested with trypsin, washed once with PBS, and then fixed in 70% ethanol overnight at 4°C. Before flow cytometry analysis, cells were stained with 200 µg/ml Ribonuclease A and 50 µg/ml propidium iodide (Sigma) for 30 min at room temperature away from light. 1×10^4^ cells per sample were analyzed using a FACSCalibur flow cytometer (BD Biosciences). Data were evaluated using Modfit software.

### Western Blot

Cells were harvested and subjected to lysis in the buffer X (50 mM Tris, 270 mM NaCl, 1% Triton X-100, pH 7.4). Equal amounts of lysates (30 µg) were separated on 10% SDS-PAGE and then transferred to polyvinylidene difluoride membranes (Millipore). Membranes were then blocked with PBS buffer containing 5% low fat milk and 0.1% Tween 20 (PBST) for 1 h at room temperature and then incubated with primary antibodies (1∶1000–2000) overnight at 4°C. After being washed three times with PBST, membranes were incubated with peroxidase-conjugated secondary antibodies (1∶5000) for 1 h at room temperature. The membranes were washed with PBST again and developed with a pro-light HRP chemiluminescence detection kit (TianGen Biotech, China). β-actin was used as a loading control.

### Statistical analysis

GraphPad Prism 5 software was used for statistical analysis. For competition binding and Guinea-pig tracheal contraction assay, the IC_50_ values were determined by using the non-linear curve-fitting program. The inhibition constants (K_i_ values) were calculated by using the Cheng-Prusoff equation. For the experiments of exogenous ACh combined with R2HBJJ, two-way ANOVA was used to compare the interaction of two treatment factors. Post-hoc analyses were performed with Bonferroni's tests for comparisons to controls. For the cell viability assay after siRNA transfection, one-way ANOVA followed by Dunnett's *t* test was used. A *P* value of less than 0.05 was considered statistically significant.
